# INF2‐ and FHOD‐related formins promote ovulation in the somatic gonad of *C. elegans*

**DOI:** 10.1002/cm.21341

**Published:** 2016-11-09

**Authors:** Anna Hegsted, Forrest A. Wright, SarahBeth Votra, David Pruyne

**Affiliations:** ^1^ Department of Cell and Developmental BiologyState University of New York Upstate Medical UniversitySyracuseNew York13210; ^2^ Department of PharmacologyState University of New York Upstate Medical UniversitySyracuseNew York13210

**Keywords:** spermatheca, reproductive defects, cell–cell junctions, actin, EXC‐6, FHOD‐1

## Abstract

Formins are regulators of actin filament dynamics. We demonstrate here that two formins, FHOD‐1 and EXC‐6, are important in the nematode *Caenorhabditis elegans* for ovulation, during which actomyosin contractions push a maturing oocyte from the gonad arm into a distensible bag‐like organ, the spermatheca. EXC‐6, a homolog of the disease‐associated mammalian formin INF2, is highly expressed in the spermatheca, where it localizes to cell‐cell junctions and to circumferential actin filament bundles. Loss of EXC‐6 does not noticeably affect the organization the actin filament bundles, and causes only a very modest increase in the population of junction‐associated actin filaments. Despite absence of a strong cytoskeletal phenotype, approximately half of ovulations in *exc‐6* mutants exhibit extreme defects, including failure of the oocyte to enter the spermatheca, or breakage of the oocyte as the distal spermatheca entrance constricts during ovulation. Loss of FHOD‐1 alone has little effect, and we cannot detect FHOD‐1 in the spermatheca. However, combined loss of these formins in double *fhod‐1;exc‐6* mutants results in profound ovulation defects, with significant slowing of the entry of oocytes into the spermatheca, and failure of nearly 80% of ovulations. We suggest that EXC‐6 plays a role directly in the spermatheca, perhaps by modulating the ability of the spermatheca wall to rapidly accommodate an incoming oocyte, while FHOD‐1 may play an indirect role relating to its known importance in the growth and function of the egg‐laying muscles. © 2016 The Authors. Cytoskeleton Published by Wiley Periodicals, Inc.

## Introduction

Formins are best known as actin filament‐nucleating proteins [Pruyne et al., 2002; Sagot et al., 2002]. Formin dimers nucleate actin filaments by stabilizing the association of two actin monomers through their actin‐binding formin homology‐2 (FH2) domains [Pruyne et al., 2002; Sagot et al., 2002; Pring et al., 2003; Moseley et al., 2004; Xu et al., 2004], with additional actin‐binding sites often provided by C‐terminal Wiskott‐Aldrich syndrome protein homology‐2 (WH2) domains or WH2‐like diaphanous autoregulatory domains (DADs) [Chhabra and Higgs, 2006; Vaillant et al., 2008; Gould et al., 2011; Heimsath and Higgs, 2012]. Proline‐rich formin homology‐1 (FH1) domains provide docking sites for the actin monomer‐binding protein profilin [Chang et al., 1997; Evangelista et al., 1997; Imamura et al., 1997; Watanabe et al., 1997], allowing formins to utilize profilin‐bound actin as a substrate for nucleation [Sagot et al., 2002; Kovar et al., 2003; Pring et al., 2003; Moseley et al., 2004]. After filament nucleation, many formins remain associated with the growing barbed end even while monomers are added, a process sometimes called processive capping [Pruyne et al., 2002; Kovar et al., 2003; Zigmond et al., 2003; Moseley et al., 2004]. During processive capping, the FH2 dimer “climbs” the barbed end [Kovar and Pollard, 2004; Mizuno et al., 2011], while the DAD sometimes enhances processivity, possibly through nonspecific electrostatic interactions [Vizcarra et al., 2014]. The FH1 domain often accelerates barbed end elongation through the rapid recruitment of profilin‐bound actin [Romero et al., 2004; Kovar et al., 2006].

However, not all formins exhibit identical biochemical properties with respect to actin filament dynamics, and some actually inhibit actin filament accumulation *in vitro*. The mammalian formin INF2 promotes actin assembly, but then severs filaments once actin‐bound ATP has been hydrolyzed and phosphate has been released [Chhabra and Higgs, 2006]. Severing by INF2 is thought to involve encircling of the actin filament by FH2 dimers to create weak points that are then exacerbated by binding of the WH2‐like DAD to adjacent subunits [Gurel et al., 2014]. Mammalian formins belonging to the FHOD subfamily also inhibit actin assembly *in vitro*, in this case through capping of the filament barbed ends [Taniguchi et al., 2009, Schönichen et al., 2013].

The free‐living nematode *Caenorhabditis elegans* provides a convenient genetic model system to study the *in vivo* functions of formins, having six formin proteins representing five of the nine conserved formin subfamilies found in animals [Mi‐Mi et al., 2012; Pruyne, 2016]. Based on FH2 domain homology, *C. elegans* encodes two INF2‐related formins: EXC‐6 (formerly known as INFT‐1) and INFT‐2 [Mi‐Mi et al., 2012]. Of these, INFT‐2 has a domain organization similar to mammalian INF2, encoding an N‐terminal diaphanous inhibitory domain (DID) that often regulates formin activity, followed by a dimerization domain and the FH1 and FH2 domains. Conversely, EXC‐6 lacks the N‐terminal DID and dimerization regions [Mi‐Mi et al., 2012]. Phenotypes have not yet been reported for worms lacking INFT‐2, but *exc‐6* mutant worms exhibit a modest locomotion defect [Mi‐Mi et al., 2012], and have defects in morphogenesis of the lumen of the excretory canal, the nematode analog of the kidney [Shaye and Greenwald, 2015]. In the excretory canal cell, EXC‐6 colocalizes with basolateral microtubules, and is suggested to link the actin and microtubule cytoskeletons. Pointing to conservation of mammalian INF2 and EXC‐6 activity, INF2 constructs bearing different DID mutations were able to partially rescue *exc‐6* mutant excretory canal defects [Shaye and Greenwald, 2015]. *C. elegans* encodes a single FHOD subfamily formin, FHOD‐1, which is important to elongation of the embryo [Vanneste et al., 2013], and the growth and maintenance of sarcomeres in the body wall muscles, and the proper function of the egg‐laying muscles [Mi‐Mi et al., 2012; Mi‐Mi and Pruyne, 2015].

We show here that EXC‐6 is expressed in the spermatheca, where it localizes to epithelial cell‐cell junctions and basolateral contractile actin filament bundles, and plays an important role in promoting ovulation. Additionally, we show that although FHOD‐1 is not detected in the spermatheca, its loss exacerbates ovulation defects in *exc‐6* mutant worms.

## Results

### Mutations of the Formin‐Coding Genes *fhod‐1* and *exc‐6* Result in Small Broods

**Figure 1:**
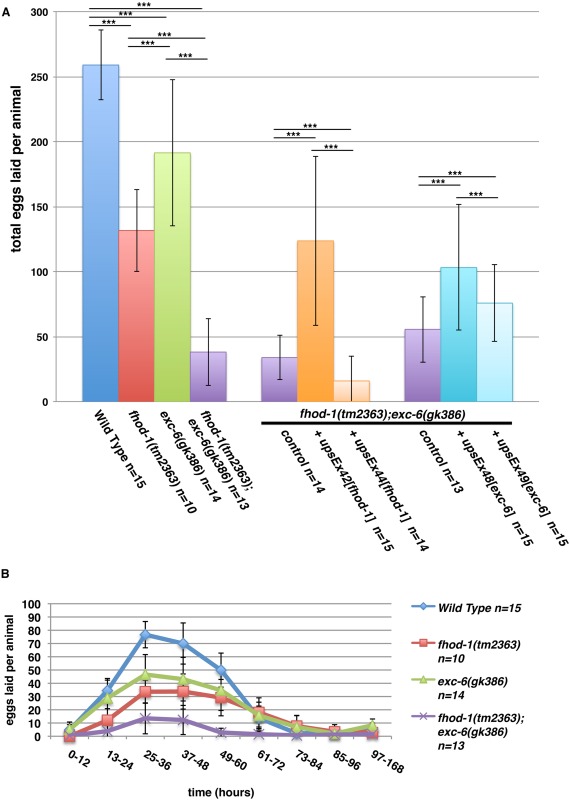
**Worms mutated for the formin genes *fhod‐1* or *exc‐6* lay fewer eggs**. (**A**) Total brood sizes. Counting eggs laid over the lifetime of hermaphrodite worms shows that the formin mutations *fhod‐1(tm2363)* and *exc‐6(gk386)* each result in reduced brood size, and their effects appear approximately additive in double *fhod‐1(tm2363);exc‐6(gk386)* mutants. Expression of wild‐type *fhod‐1* from the extrachromosomal array *upsEx42*, or of wild‐type *exc‐6* from the arrays *upsEx48* or *upsEx49*, partially rescues this defect. **(B)** Timing of egg laying. Counting eggs laid per animal over the indicated time intervals demonstrates that formin mutants do not cease egg laying prematurely, but have a slower rate of egg production throughout their fertile period. Results shown are representative of three independent experiments. Bars indicate mean number of eggs counted for *n* animals, and error bars indicate one standard deviation. *** indicates *P* < 0.001.

In an earlier study of *C. elegans* formins, we had found that hermaphrodite worms bearing the deletion allele *fhod‐1(tm2363)*, which disrupts the FH2 domain of the formin FHOD‐1, develop smaller body wall muscles and lay fewer eggs than wild type [Mi‐Mi et al., 2012]. As part of that study, we also combined *fhod‐1(tm2363)* with *exc‐6(gk386)*, a mutation that eliminates the only known start site for a second formin‐coding gene, *exc‐6* (previously called *inft‐1*). Although we noted in that study that mutation of *exc‐6* caused no additional defects in body wall muscles, we report here that *fhod‐1(tm2363);exc‐6(gk386)* double mutants produce very small broods, laying roughly 15% the number of eggs as wild‐type hermaphrodites (Fig. 1A). We had previously shown that *fhod‐1(tm2363)* single mutants also lay approximately 50% fewer eggs than wild type [Mi‐Mi et al., 2012], but also observed that *exc‐6(gk386)* single mutant hermaphrodites lay fewer eggs than wild type (Fig. 1A). Thus, EXC‐6 and FHOD‐1 each contribute to producing a normal brood size.

In order to verify that the formin gene mutations were the cause of these defects, we knocked down expression of *fhod‐1*, *exc‐6*, or both, using RNAi in animals that were otherwise wild type for formins. Again, we observed small brood sizes of treated animals compared to animals subjected to control RNAi treatment (Fig. S1A, Supporting Information). We also tested whether exogenous FHOD‐1 or EXC‐6 could rescue these defects in *fhod‐1(tm2363);exc‐6(gk386)* mutant hermaphrodites by introducing wild‐type *fhod‐1* or *exc‐6* genes as extrachromosomal arrays. Although gene expression and heritability from extrachromosomal arrays can be variable [Mello and Fire, 1995] and the different arrays had varying effects, both formin genes partially rescued the small brood size of double mutants in at least one transformed line (Fig. 1A).

In the absence of male worms, *C. elegans* hermaphrodites produce eggs through self‐fertilization. Hermaphrodites produce sperm during late larval development, and store them during adulthood in the bag‐like spermathecae. Hermaphrodites utilize those sperm to fertilize oocytes that pass through the spermathecae during ovulation, until the sperm are depleted. One possible cause of a small brood size is the production of fewer sperm, which would result in a truncated period of egg‐laying. However, formin mutant hermaphrodites laid eggs over a very similar time course as wild type, but with mutants laying consistently fewer eggs at all times (Fig. 1B). Similar results were found after RNAi treatment against *fhod‐1* and *exc‐6* (Fig. S1B, Supporting Information). A direct count of sperm in the spermathecae of young adult mutant and wild‐type hermaphrodites showed similar numbers, also arguing against a defect in sperm production. Thus, FHOD‐1 and EXC‐6 promote production of fertilized eggs, but not through spermatogenesis.

### *exc‐6* and *fhod‐1* are Not Required for Normal Gonad Morphogenesis

**Figure 2:**
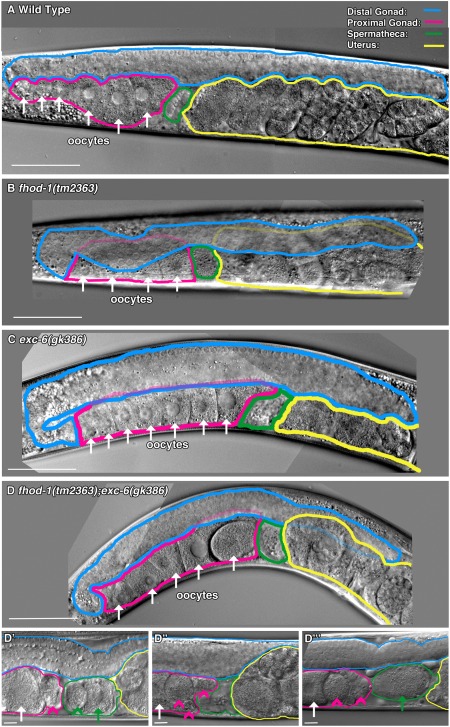
**Gonad morphology is grossly normal in the absence of FHOD‐1 or EXC‐6. (A‐D) DIC microscopy showing half of the hermaphrodite gonad, including one gonad arm.** Examples are shown of (A) wild type, (B) *fhod‐1(tm2363)*, (C) *exc‐6(gk386)*, and (D) *fhod‐1(tm2363);exc‐6(gk386)* animals. For all strains, gonads appear grossly normal, with long distal arms (*blue*) containing immature germ cells, proximal arms (*pink*) containing oocytes (*arrows*), and spermathecae (*green*) adjacent to uteruses (*yellow*) containing developing embryos. **(D‐D″′) Double mutant worms have cellular fragments in the proximal gonad**. Specific to the *fhod‐1(tm2363);exc‐6(gk386)* gonads, abnormal cell fragments are visible in the proximal gonad (*pink arrowheads*) or spermatheca (*green arrowhead*). Green arrows indicate oocytes in the spermatheca being fertilized. Scale bars, 50 µm.

To determine whether gross morphological defects of the gonad could account for low brood sizes of the formin mutants, we compared overall gonad morphology of young adult mutant and wild‐type hermaphrodites. When observed using DIC microscopy, the gonads of worms bearing *fhod‐1(tm2363)*, *exc‐6(gk386)*, or both mutations resembled wild type, with two gonad arms attached to a central uterus. Figure 2 shows examples of half of the symmetric hermaphrodite gonad, including one gonad arm and part of the uterus. Like wild‐type animals, mutant hermaphrodites have small developing germ cells in the distal portion of their gonad arms (Fig. 2, *blue*), larger oocytes lined up in the proximal portion (Fig. 2, *pink*), small indistinct‐appearing sperm in the spermatheca (Fig. 2, *green*), and developing embryos in the uterus (Fig. 2, *yellow*). Only *fhod‐1(tm2363);exc‐6(gk386)* double mutants exhibited frequent aberrations visible by DIC microscopy, in the form of fragments of oocytes in their proximal gonad arms (Fig. 2D'‐D''', *arrowheads*).

**Figure 3:**
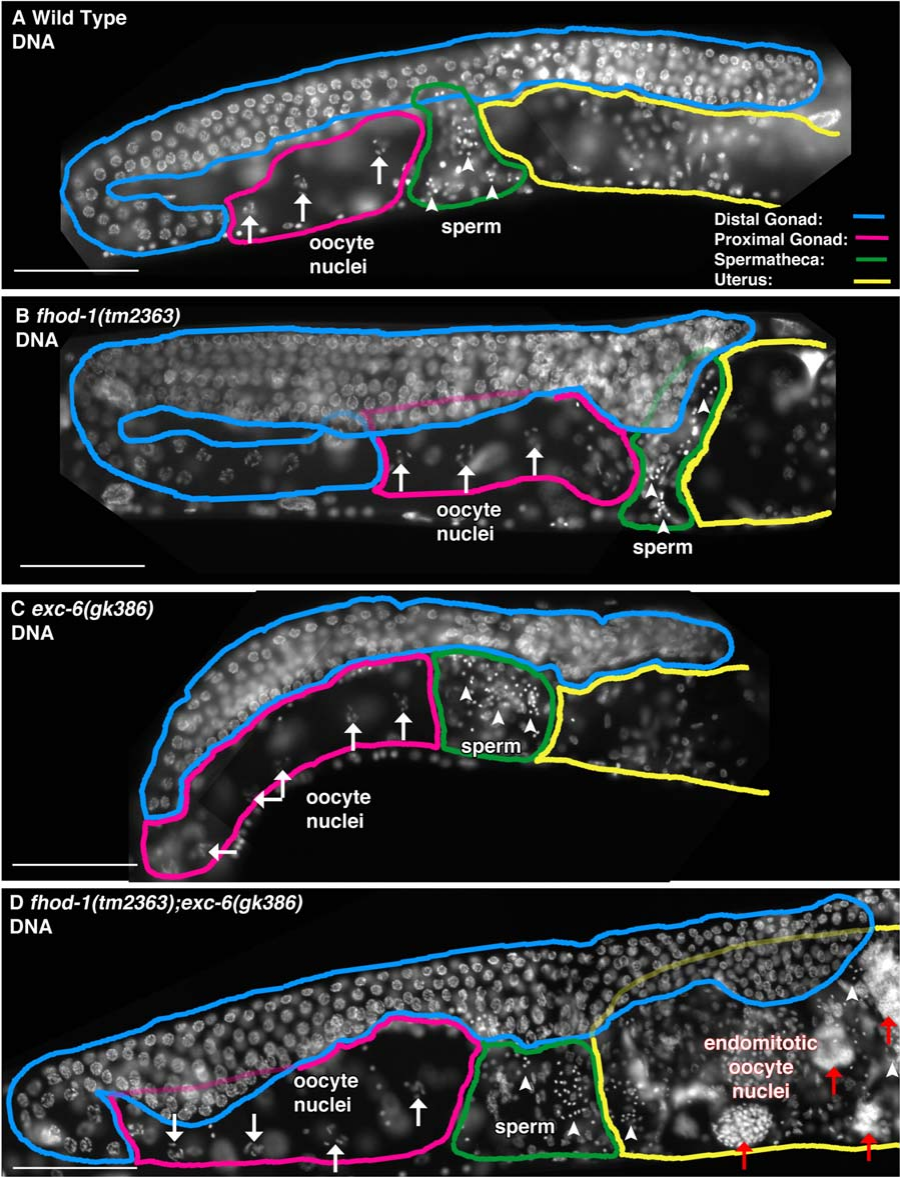
**Endomitotic oocytes form in the simultaneous absence of FHOD‐1 and EXC‐6**. **(A‐D)** Nuclei in the region of one gonad arm were visualized by fluorescence microscopy of DAPI‐stained animals. Examples are shown of (A) wild type, (B) *fhod‐1(tm2363)*, (C) *exc‐6(gk386)*, and (D) *fhod‐1(tm2363);exc‐6(gk386)* animals. For all strains, abundant nuclei of immature germ cells populate the distal gonad arms (*blue*), condensed chromosomes are visible in oocyte nuclei (*white arrows*) in the proximal gonad arms (*pink*), and punctate sperm nuclei (*white arrowheads*) are present in the spermathecae (*green*) adjacent to the uterus (*yellow*). Unique for the double mutants, large DAPI‐stained masses that are present in the proximal gonad or the uterus (*red arrows*) are likely the nuclei of endomitotic oocytes. Scale bars, 50 µm.

As a second method of inspecting gonad organization, we examined nuclei through DAPI staining. In the gonad, the overwhelming majority of nuclei are from germ cells or fertilized embryos. DAPI stain revealed for the wild type and for all mutant strains: normal‐appearing immature germ cell nuclei in the distal gonad (Fig. 3, *blue*), oocyte nuclei with condensed chromosome pairs (*arrows*) in the proximal gonad (*pink*), highly condensed sperm nuclei (*arrowheads*) in the spermatheca (*green*), and embryos with varying numbers of nuclei in the uterus (*yellow*). However, we also observed large masses of DNA (Fig. 3D, *red arrows*) in the proximal gonad and/or uterus of 50% ± 4.5% of double mutant hermaphrodites (n ≥ 28 animals from two independent experiments). These are likely endomitotic oocytes that were activated for ovulation, but not fertilized in a timely manner, resulting in mitosis without cytokinesis [Iwasaki et al., 1996].

### *exc‐6* and *fhod‐1* Promote Normal Ovulation

**Figure 4:**
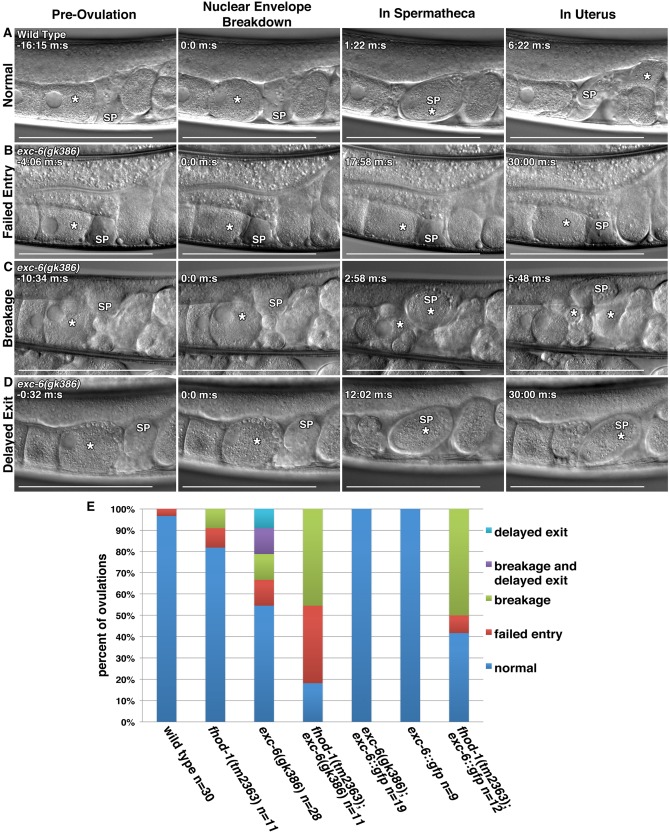
**EXC‐6 and FHOD‐1 promote ovulation**. (**A‐D**) Ovulation was visualized in live worms using DIC microscopy. In all images, (*) indicates the ovulating oocyte or fertilized embryo, and (SP) indicates the spermatheca. (A) Normal ovulation can be subdivided into four landmarks: *Pre‐ovulation* when the proximal oocyte has an intact nucleus, *Nuclear Envelope Breakdown* (NEBD) and coincident rounding of the proximal oocyte, In *Spermatheca*, when ovulation has completed and the oocyte has entered the spermatheca, and In *Uterus*, when the fertilized embryo (*) has exited the spermatheca to the uterus. In three examples of defective ovulations in *exc‐6(gk386)* hermaphrodites, we observe: (B) *Failed Entry* of the proximal oocyte into the spermatheca following NEBD, (C) *Breakage* of the proximal oocyte into two pieces (two asterisks) during its entry into the spermatheca, and (D) *Delayed Exit*, in which the fertilized embryo fails to exit the spermatheca within 30 min. Scale bars, 100 µm. Time stamps indicate min:sec. (**E**) Frequencies of ovulations phenotypes. While ovulations of wild‐type worms were almost always normal, less that 20% of *fhod‐1(tm2363);exc‐6(gk386)* double‐mutant ovulations were normal. Only 50% of *exc‐6(gk386)* ovulations were normal. An *exc‐6::gfp* transgene fully rescued the *exc‐6(gk386)* defects, had no effect in a wild‐type background, but severely exacerbated ovulation in a *fhod‐1(tm2363)* background. Shown are the combined results of two independent experiments.

The formation of endomitotic oocytes as we observed in the *fhod‐1(tm2363);exc‐6(gk386)* double mutants has been tied to defects in ovulation and the activity of the somatic cells that line the gonad [Iwasaki et al., 1996]. To probe for ovulation defects, we used DIC microscopy to monitor the gonads of wild‐type and mutant hermaphrodites during ovulation. The first observable indication of ovulation is nuclear envelope breakdown (NEBD) and rounding up of the oocyte proximal to the spermatheca (Fig. 4A) [McCarter et al., 1997, 1999]. While this is occurring, myoepithelial sheath cells lining the proximal gonad arm contract, pushing the oocyte into through the distal opening of the spermatheca as it dilates. Once ovulation has completed and the oocyte is inside the spermatheca, the distal spermatheca contracts, the oocyte is rapidly fertilized, and the fertilized embryo is expelled into the uterus [McCarter et al., 1997, 1999]. We observed normal ovulations in wild‐type animals almost every time (Fig. 4A, E). Conversely, nearly 50% of ovulations in *exc‐6(gk386)* animals exhibited one or more of three distinct defects (Fig. 4B‐E). These defects included failed ovulations, in which the distal spermatheca did not dilate and the activated oocyte was not able to enter the spermatheca (Fig. 4B, E, *Failed Entry*), breakage of the oocyte into two pieces as it entered the spermatheca during ovulation (Fig. 4C, E, *Breakage*), and a very rare post‐ovulation failure of the fertilized egg to exit the spermatheca for the duration of the 30 min imaging session (Fig. 4D, E, *Delayed Exit*). Mutation of *fhod‐1* alone had relatively little effect, resulting in > 80% normal ovulations (Fig. 4E), but *fhod‐1(tm2363)* further exacerbated ovulation defects when combined with *exc‐6(gk386)*, with < 20% ovulations occurring normally in double mutants (Fig. 4E, Fig. S2, Supporting Information).

**Figure 5:**
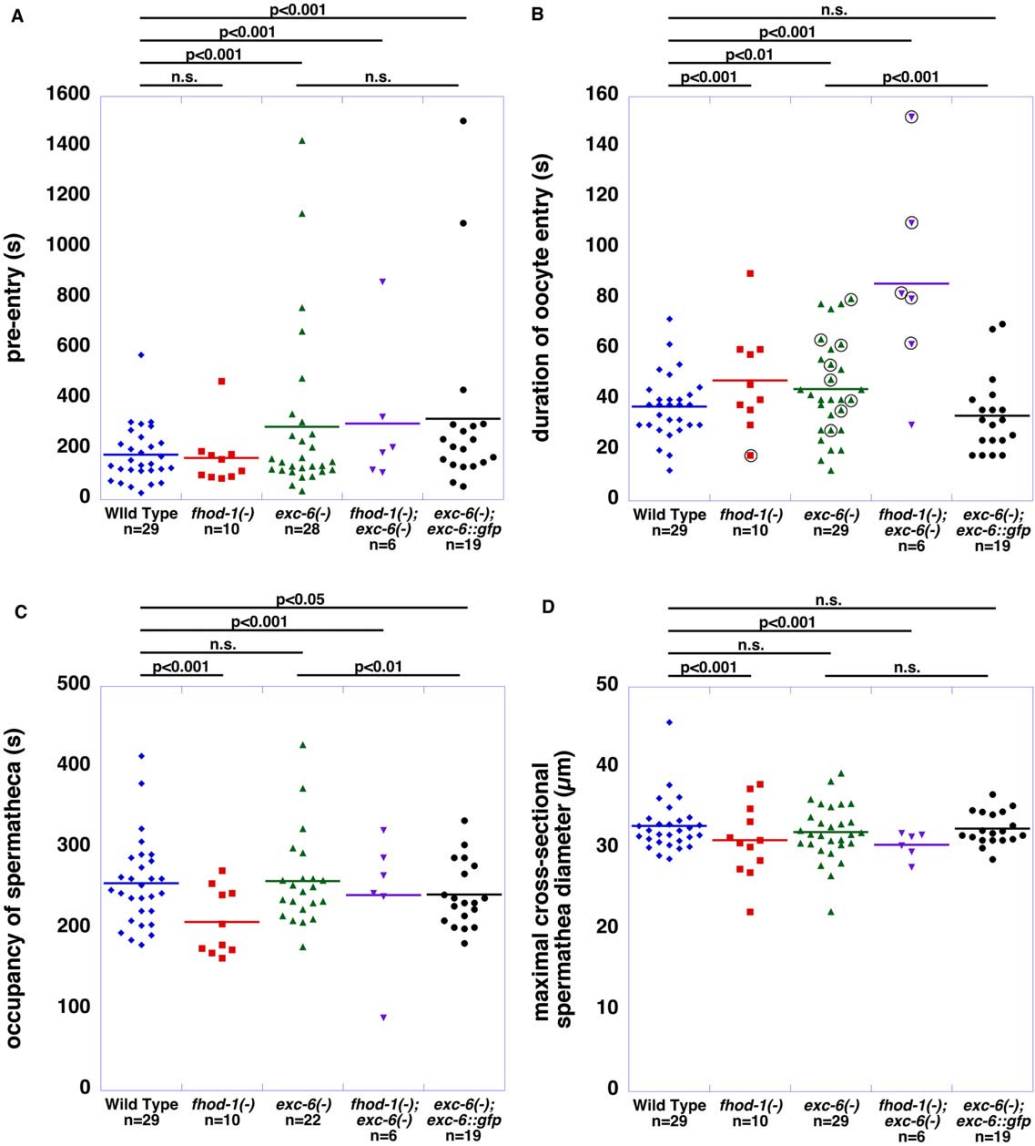
**FHOD‐1 and EXC‐6 affect the timing of different ovulation events**. (**A**) Time between oocyte activation and the start of oocyte entry into the spermatheca (pre‐entry). The start of entry was not affected by *fhod‐1(tm2363)*, but was delayed by *exc‐6(gk386)*, and this was not rescued by *exc‐6::gfp*. (**B**) Duration of entry of the oocyte into the spermatheca. The time from the start of oocyte entry to its completion was extended for both *fhod‐1(tm2363)* and *exc‐6(gk386)* mutants, and this was exacerbated in double mutant worms. The effect of *exc‐6(gk386)* on this was rescued by *exc‐6::gfp*. Events in which oocytes were broken during entry (*circled data points*) did not correlate with short periods of entry. (**C**) Time of occupancy of the spermatheca by the fertilized embryo. Excluding highly abnormal ovulations in which the fertilized embryo is trapped in the spermatheca for a prolonged period (which were not included here), the duration of spermatheca occupancy by the fertilized embryo was unaffected by *exc‐6(gk386)*, but decreased by *fhod‐1(tm2363)*. (**D**) Maximal cross‐sectional diameter of the spermatheca during oocyte entry. The formin mutations did not affect the degree to which the spermatheca distends when an oocyte enters the spermatheca. For (A‐D), dots represent individual ovulation events, bars indicate the mean, and *n* = number of ovulations viewed. Statistical significances are indicated, with n.s. indicating not significant. Shown are the combined results of two independent experiments.

The formin mutations also perturbed the timing of events during ovulation. Mutation of *exc‐6*, but not *fhod‐1*, was associated with delays in the initiation of entry of the activated oocyte into the spermatheca (Fig. 5A). Once entry was initiated, mutants for either formin displayed slowed rates of oocyte entry, with double mutants being significantly slower than wild type in nearly every ovulation observed (Fig. 5B). Notably, the ovulations in which oocytes were broken (Fig. 5B, *circled data points*) were generally those with longer periods of entry, arguing against a premature contraction of the distal spermatheca opening as the cause of this phenotype. Ignoring the rare prolonged trapping of fertilized eggs in the spermatheca, *exc‐6(gk386)* did not alter the duration of occupancy of the spermatheca after ovulation by the fertilized egg, while *fhod‐1(tm2363)* reduced this time, accelerating exit of the egg from the spermatheca (Fig. 5C). The phenotypes of failed and slow entry of oocytes into spermathecae in formin mutants suggested that the walls of mutant spermathecae might be unable to relax at the normal rate. However, the spermathecae of wild type and formin mutants eventually distend to virtually identical degrees after oocyte entry, as measured by their maximal cross‐sectional diameters (Fig. 5D). Thus, *exc‐6* appears important for the prompt initiation of oocyte entry during ovulation, both *fhod‐1* and *exc‐6* appear to promote efficient movement of the oocyte into the spermatheca once entry is initiated, and *fhod‐1* plays a role in the timing of the exit of the fertilized embryo from the spermatheca.

To test whether the ectopic EXC‐6 could rescue these ovulation defects, we stably introduced into the genome an *exc‐6* transgene driven by the *exc‐6* promoter, and engineered to include a C‐terminal GFP tag. The biolistic bombardment method of transformation used did not replace the endogenous *exc‐6* locus, but introduced one or more copies of the transgene to a single random location in the genome [Praitis et al., 2001]. Notably, *exc‐6::gfp* completely rescued the gross ovulation abnormalities of *exc‐6(gk386)* mutants when crossed into that background (Fig. 4E), and also rescued the slow rate of oocyte entry into the spermatheca (Fig. 5B). However, it was unable to rescue the delay in the initiation of entry of the activated oocyte (Fig. 5A), and caused a very modest decrease in the time spent by oocytes in the spermatheca (Fig. 5C). Worms with wild‐type *exc‐6* and also expressing *exc‐6::gfp* ovulated normally, demonstrating that EXC‐6 overexpression does not negatively affect ovulation in an otherwise wild‐type background. In contrast, *exc‐6::gfp* exacerbated the ovulation defects observed in *fhod‐1(tm2363)* hermaphrodites, increasing the frequency of aberrant ovulations to > 50% (Fig. 4E), suggesting the two formins are not interchangeable in their contributions to ovulation. Unfortunately, an integrated *fhod‐1::gfp* transgene that partially rescues body wall muscle development in *fhod‐1(tm2363*) mutant worms [Mi‐Mi et al., 2012] was unable to rescue their small brood size (Fig. S3A,B, Supporting Information). Considering that an untagged *fhod‐1* transgene was able to provide rescue (Fig. 1A), it seems likely that C‐terminal GFP interferes with FHOD‐1 function in this process.

### EXC‐6::GFP Localizes to Cell‐Cell Junctions and Contractile Actin Filament Bundles in the Spermatheca

**Figure 6:**
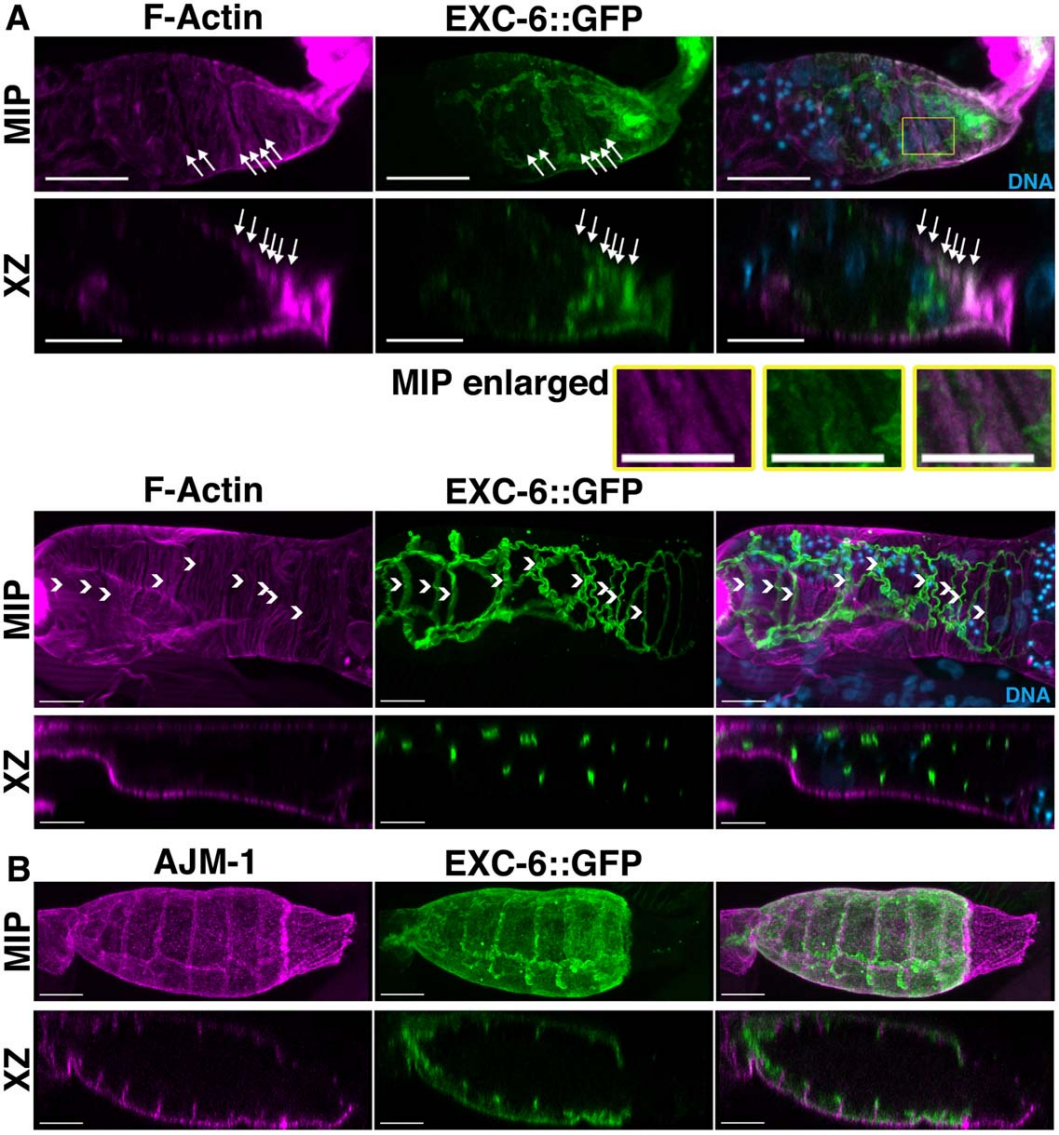
**EXC‐6::GFP is expressed in the spermatheca and localizes to cell‐cell junctions and contractile actin filament bundles**. Shown are maximum intensity projections (MIP) and XZ slices (XZ) reconstructed from confocal Z‐stacks. **(A)** Spermathecae of EXC‐6::GFP expressing animals were stained with DAPI to mark DNA and fluorescent phalloidin to mark F‐actin. EXC‐6::GFP localizes to F‐actin bundles near the basal surfaces of the epithelial cells lining the spermatheca (*arrows* in top spermatheca), and to wavy ribbons closer to the spermatheca lumen (*arrowheads* in bottom spermatheca). The boxed region of the top spermatheca is enlarged (MIP enlarged) to highlight F‐actin bundles with faint associated EXC‐6::GFP. (**B**) Spermathecae of EXC‐6::GFP‐expressing animals were immunostained for AJM‐1. AJM‐1 decorates spermatheca apical and lateral junctions, and EXC‐6::GFP overlaps with the apical portion of AJM‐1. Scale bars, 10 µm.

**Figure 7:**
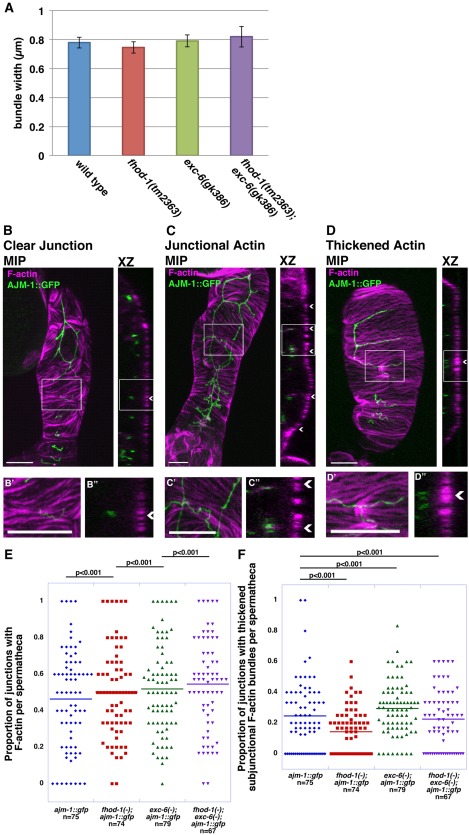
**F‐actin is largely normal in *exc‐6* and *fhod‐1* mutants**. (**A**) Widths of F‐actin bundles. The width of basal F‐actin bundles in spermathecae do not differ statistically between wild type, *fhod‐1(tm2363), exc‐6(gk386)* and *fhod‐1(tm2363);exc‐6(gk386)*. Data shown are the average of two independent experiments (*n* = 120–125 actin filament bundles from 12‐14 spermathecae per strain). **(B‐D)** Spermathecae of AJM‐1::GFP expressing animals were stained with fluorescent phalloidin to mark F‐actin. Maximum intensity projections (MIP) and XZ slices (XZ) reconstructed from confocal Z‐stacks are shown. Magnified views of boxed sections of MIPs (B'‐D') and XZ slices (B''‐D'') are also shown. Examples include junctions (*arrowheads*) with no visible associated F‐actin (B‐B''), junctions with associated F‐actin (C‐C''), and junctions with adjacent thickened F‐actin bundles at the basal surface (D‐D''). Scale bars, 10 µm. **(E)** Frequency of visible junction‐associated F‐actin. Worms with the single or double formin mutations have a slightly higher proportion of junctions with F‐actin than wild type. **(F)** Frequency of thickened F‐actin bundles adjacent to junctions. Worms with the *exc‐6(gk386)* mutation have a higher proportion of junctions with thickened adjacent F‐actin bundles than wild‐type animals, while *fhod‐1(tm2363)* mutants have lower, and *fhod‐1(tm2363);exc‐6(gk386)* double mutants have nearly wild‐type proportions. (E, F) are each the combined data of four independent experiments. Bars indicate mean values, and n indicates the number of spermathecae analyzed, with 2 to 13 visible junctions scored per spermatheca.

We were not able to successfully visualize endogenous EXC‐6 by immunofluorescence microscopy. However, we did observe strong expression of the rescuing *exc‐6::gfp* transgene (driven by the *exc‐6* promoter) in the epithelial cells that make up the wall of the spermatheca (Fig. 6), but not in other parts of the somatic gonad, or in the germline. These epithelial cells are notable for very prominent contractile actin filament bundles situated at their basal surfaces, and oriented circumferentially around the spermatheca [Strome, 1986]. Consistent with the ability of formins to interact with actin filaments, fluorescent phalloidin staining revealed some EXC‐6::GFP was associated with these actin filament bundles (Fig. 6A, *arrows*). Even more prominent, however, were ribbons of EXC‐6::GFP near the spermatheca lumen (Fig. 6A, B), a localization resembling those of known markers of cell‐cell junctions. Confirming this, immunostaining for AJM‐1 [Podbilewicz and White, 1994], which localizes to apical and lateral junctions of the spermatheca [Köppen et al., 2001; Lints and Hall, 2009], showed EXC‐6::GFP is associated with spermatheca cell‐cell junctions, but at a position slightly more apical relative to AJM‐1 (Fig. 6C). Note, compared to the fixation conditions used to preserve F‐actin for phalloidin staining, the methanol‐based fixation used here for immunostain enhanced the preservation of the basal surface‐associated EXC‐6::GFP, while junctional EXC‐6::GFP appears relatively enhanced in the phalloidin‐stained images. The same explanation would account for similar differences between the localizations of AJM‐1 in Fig. 6B, and AJM‐1::GFP in Fig. 7B‐D.

We also probed for FHOD‐1 in the somatic gonad, but found no evidence by immunostaining with antibodies that recognize endogenous FHOD‐1 in other worm tissues, or by examining four different FHOD‐1::GFP‐expressing strains (Fig. S4, Supporting Information and A. Hegsted, unpublished data). Rather, the only association with the gonad that we observed for FHOD‐1 was a strong presence in the egg laying muscles, as previously reported [Mi‐Mi et al., 2012].

### The F‐Actin Cytoskeleton Is Relatively Normal in the Spermathecae of *exc‐6* and *fhod‐1* Mutants

To determine whether actin filament organization in the spermatheca was perturbed in the formin mutants, we compared fluorescent phalloidin‐stained spermathecae of wild‐type and mutant animals. Overall, spermatheca F‐actin organization appeared relatively normal in both single mutants and in the *fhod‐1(tm2363);exc‐6(gk386)* double mutant, when observed in intact animals. However, when spermathecae were dissected from worms for more detailed analysis, we found that basal F‐actin bundles were sometimes disrupted in the spermathecae of *fhod‐1(tm2363)* animals (3 of 86 spermathecae) and of *fhod‐1(tm2363);exc‐6(gk386)* animals (8 of 77 spermathecae), but never in wild type (of 75 spermathecae) and only once (out of 80 spermathecae) in *exc‐6(gk386)* worms, suggesting the actin filament bundles might be somewhat more labile in the absence of FHOD‐1. On inspection of intact actin filament bundles, we measured no consistent differences in the intensity of phalloidin stain (A. Hegsted, unpublished data), or in the widths of the bundles (Fig. 7A), suggesting the amount of filamentous actin in these structures does not differ between wild‐type and formin mutant spermathecae.

Considering the strong localization of EXC‐6::GFP to spermatheca cell‐cell junctions, we also searched for junction‐associated F‐actin in the wild‐type and formin mutant strains. To assist in this, we crossed a transgene expressing GFP‐tagged AJM‐1 as a marker for the junctions [Podbilewicz and White, 1994], into the *fhod‐1(tm2363)*, *exc‐6(gk386)*, and *fhod‐1(tm2363);exc‐6(gk386)* backgrounds. We observed a small amount of F‐actin along a subset of junctional surfaces in the spermathecae, particularly near the apical regions (compare Fig. 7B‐B'' and 7C‐C''). The qualitative appearance of junctional F‐actin did not noticeably differ between wild‐type and formin mutant spermatheca, although the proportion of junctions with a detectable amount of associated F‐actin increased very slightly in each of the formin mutants (Fig. 7E). Another F‐actin structure we observed associated with a subset of junctions in the spermathecae was thickened basal F‐actin bundles immediately adjacent to the junction (Fig. 7D‐D''). The proportion of spermatheca junctions with this thickened subjunctional F‐actin was very modestly higher in *exc‐6(gk386)* animals, but reduced in *fhod‐1(tm2363)* animals, and nearly normal in the double *fhod‐1(tm2363);exc‐6(gk386)* animals (Fig. 7F). Thus, neither formin appeared to be necessary for the assembly of F‐actin in the circumferential contractile bundles or at the cell‐cell junctions, but the frequency at which F‐actin was observable at the junctions was very slightly increased in each of the formin mutants.

## Discussion

In the *C. elegans* hermaphrodite, the production of fertilized eggs depends on ovulation, during which activation of an oocyte is coupled to the transfer of the oocyte from the proximal gonad into a bag‐like spermatheca, where it is rapidly fertilized by a resident sperm [McCarter et al., 1999]. This movement is controlled by contractions of an actomyosin meshwork in myoepithelial sheath cells lining the proximal gonad, which push the activated oocyte through the dilated valve leading to the spermatheca. After entry of the oocyte into the spermatheca, contractions of circumferential actin filament bundles in the epithelial cells lining the spermatheca close this valve, and expel the now‐fertilized embryo through the spermatheca‐uterine valve into the uterus.

A notable feature of this process is the dramatic shape changes that the spermatheca undergoes, dramatically stretching on entry of an oocyte, followed by retraction after expulsion of the fertilized egg (Fig. 4A). To facilitate these changes, the epithelial cells lining the spermatheca have unusual cell‐cell junctions with three components. Smooth septate junctions, considered to be the functional equivalents of vertebrate tight junctions, contain Discs large (DLG‐1) and the worm‐specific apical junction marker AJM‐1 [Köppen et al., 2001]. These junctions span much of the lateral portions of the spermatheca junctions, and are interspersed with gap junctions [Lints and Hall, 2009]. Apical to this layer are adherens junctions, which resemble their vertebrate counterparts by containing cadherin–catenin complexes, and being associated with actin filament bundles [Costa et al., 1998; Köppen et al., 2001; McMahon et al., 2001]. Finally, unique to the spermatheca is a layer of pleated septate junctions directly adjacent to the lumen, which unfold and refold in an accordion‐like manner to accommodate the stretch and contraction of the spermatheca during and following ovulation [Lints and Hall, 2009].

We show here that two members of the actin‐organizing formin family, FHOD‐1 and EXC‐6, are important for normal ovulation. Worms bearing a deletion allele of the *exc‐6* gene showed variable defects during ovulation, including delayed or outright failed entry of activated oocytes into the spermatheca, and the breakage of many oocytes during their entry into spermatheca (Figs. 4 and 5). Although a deletion mutation of *fhod‐1* alone had little effect on ovulation, combination of the *fhod‐1* and *exc‐6* mutations greatly increased the incidence of defective ovulations (from 50% to 80%), and significantly slowed the passage of oocytes into the spermatheca (Figs. 4 and 5).

Although we were unable to visualize endogenous EXC‐6, a rescuing EXC‐6::GFP fusion protein driven by the *exc‐6* promoter was expressed in the epithelial cells that line the spermatheca (Fig. 6), but was not detectable in other parts of the gonad, including the myoepithelial sheath cells or in oocytes. We were unable to observe either endogenous FHOD‐1 or FHOD‐1::GFP fusion proteins in any part of the somatic gonad (Fig. S4, Supporting Information), but this does not rule out the possibility of a FHOD‐1 population below our level of detection. Within the spermatheca, EXC‐6::GFP localized along the circumferential actin filament bundles situated near the basal surfaces (Fig. 6A). Additionally, EXC‐6::GFP appeared along the cell‐cell junctions, at a position that is apical relative to the smooth septate junction marker AJM‐1 (Fig. 6B), suggesting the formin is associated with the adherens and/or pleated septate junctions.

Despite the fact that many formins have been tied to the assembly of actin filamentous structures, the circumferential actin filament bundles and the junctional F‐actin appeared normal in the spermatheca even in the *fhod‐1;exc‐6* double mutant. It may be significant that the mammalian homologs of FHOD‐1 and EXC‐6 are distinct from many other formins in their interactions with actin. The EXC‐6‐related formin INF2 is notable for severing actin filaments *in vitro* [Chhabra and Higgs, 2006], while FHOD‐1‐related formins FHOD1 and FHOD3 inhibit barbed end elongation [Taniguchi et al., 2009; Schönichen et al., 2013]. Thus, FHOD‐1 and EXC‐6 might alter actin filament organization in a manner not readily apparent through light microscopy, such as limiting filament length through severing or capping activity. Such a mechanism could possibly account for a very slight increase in the proportion of junctions with detectable F‐actin in the formin mutants relative to wild type (Fig. 7E).

A variety of genes that have been shown to regulate spermatheca function are involved in controlling the organization of the circumferential actin filament bundles, or in controlling the calcium signaling that regulates their contraction. Oocyte entry into the spermatheca initiates calcium signaling through phospholipase C‐ε (PLC‐1), which produces inositol 1,4,5‐trisphosphate (IP_3_), which triggers the IP_3_ receptor (ITR‐1) to release calcium from the endoplasmic reticulum [Kariya et al., 2004; Baylis and Vázquez‐Manrique, 2012]. Calcium waves propagate from cell to cell through gap junctions, activating non‐muscle myosin (NMY‐1) and promoting spermatheca contraction in a manner facilitated by the actin filament‐bundling protein filamin (FLN‐1) [Kovacevic and Cram, 2010; Kovacevic et al., 2013]. However, disruption of many components of this pathway do not lead to defects in ovulation, but generally result in trapping of the fertilized egg in the spermatheca, something only very rarely seen during *exc‐6* mutant ovulations (Fig. 4E).

Defects in a host of other cytoskeletal components do lead to ovulation defects similar to the formin mutants (failed entry and broken oocytes), including mutation or knock‐down of genes encoding tropomyosin (*lev‐11*), members of the troponin complex (TnC *pat‐10*, TnT *mup‐2*, TnI *tni‐1*, *unc‐27/tni‐2*), paramyosin (*unc‐15*), muscle myosin heavy chains (*myo‐3*, *unc‐54*), nonmuscle myosin heavy and light chains (*nmy‐2* and *mlc‐4*), β‐integrin (*pat‐3*), vinculin (*deb‐1*), and AIP1 (*unc‐78*, *aipl‐1*) [Myers et al., 1996; Ono and Ono, 2004; Ono et al., 2007; Obinata et al., 2010; Ono and Ono, 2014; Ono and Ono, 2016]. However, these have all been implicated in regulating the contractions of the myoepithelial sheath cells, rather than defects of spermatheca function. An exception to this is the homolog of the actin filament‐severing protein flightless, encoded by *fli‐1*, which is highly expressed in the spermatheca, and whose loss results in similar ovulation defects as the formin mutants [Deng et al., 2007].

A plausible spermatheca‐dependent mechanism for the ovulation failures seen in the formin mutants is a reduced ability of the spermatheca wall to stretch rapidly. The association of EXC‐6::GFP with the junctions would be consistent with such a possibility, as normal adherens junction function is important for morphological changes in many epithelia [Gumbiner, 1996; Tepass, 1999], and pleated septate junctions are thought to be critical for spermatheca stretch. An alternative and not mutually exclusive possibility is that EXC‐6 might affect calcium‐based signaling through gap junctions, which coordinates contractions across the spermatheca [Kovacevic et al., 2013]. Reduced ability of the distal spermatheca to dilate would explain ovulations in which activated oocytes fail to enter the spermatheca (Fig. 4B,E). In the cases where oocytes do enter the spermatheca, formin mutant spermathecae ultimately stretch to the same extent as wild type, but they appear to do so more slowly, prolonging the time it takes for oocytes to completely enter the spermatheca (Fig. 5B). This slowed entry is particularly notable in *fhod‐1;exc‐6* double mutants, and may at least partially explain the breakage of oocytes, which tend to occur during ovulation events in which entry is prolonged (Fig. 5B, *circled data points*). It is not clear how the formins might cause such a slowing. One possibility is that improperly organized junctional actin filaments mechanically inhibit changes in junction shape, or could disrupt gap junction‐dependent cell‐cell coordination.

While the expression of EXC‐6::GFP in the spermatheca suggests this formin plays a direct role, the absence of detectable FHOD‐1 in the somatic gonad leaves open the possibility that this formin contributes to ovulation in an indirect manner. We had previously shown that FHOD‐1 is expressed in the egg‐laying muscles, and that in *fhod‐1* mutants these muscles are deficient at egg‐laying in response to pharmacological stimulation with serotonin [Mi‐Mi et al., 2012]. Moreover, embryos within the uterus of *fhod‐1* mutants are on average older than those in wild‐type animals, indicating a longer dwell time in the uterus, possibly due to a partial defect in egg‐laying. Despite this, *fhod‐1* mutant worms accumulate the same number of embryos in their uterus as wild‐type animals [Mi‐Mi et al., 2012]. This suggests the existence of a feedback mechanism, whereby a full uterus produces signals that inhibit ovulation. Supporting this, when young adult hermaphrodites are examined, *fhod‐1* mutants are less likely to be experiencing an ovulation at any one instant than wild type (22.5% ± 5.8% of observed *fhod‐1* mutant worms are ovulating, vs. 33.4% ± 2.6% of wild‐type worms).

At this point, we can only speculate on how an indirect role for FHOD‐1 could lead to the genetic interactions we observed between the formins. In the case of the increased severity of ovulation defects in *fhod‐1;exc‐6* double mutants, it is possible that inhibitory signals due to loss of FHOD‐1 adversely affect an ovulation process that is already compromised by the absence of EXC‐6. It is more difficult to imagine a mechanism that would explain why overexpression of EXC‐6 from the *exc‐6::gfp* transgene would be detrimental in a *fhod‐1* mutant. One speculative possibility is that excess EXC‐6 might override putative inhibitory signals in *fhod‐1* mutants, leading to overstuffing of the spermatheca and uterus, and slowed entry of oocytes into the spermatheca during ovulation. This could account for the increased breakage of oocytes during ovulation seen in *fhod‐1;exc‐6::gfp* animals. A broader genetic analysis of interactions between *exc‐6*, *fhod‐1*, and other regulators of ovulation will be important to better understand the complex contribution of these formins to this process.

## Materials and Methods

### Plasmids

The genomic sequence of *exc‐6*, including 5 kb upstream and 2 kb downstream, was obtained from the yeast artificial chromosome *Y11F11* (provided by the Wellcome Trust Sanger Institute, Cambridge, UK) and subcloned into pRS315 [Sikorski and Hieter, 1989] using the Gene Cloning and Tagging of *C. elegans* Genes using Yeast Homologous Recombination (GeneCATCHR) method [Sassi et al., 2005] in the yeast strain ASHSY2 (a gift from A. Spence, University of Toronto, Toronto, Ontario), resulting in the plasmid pRS315‐*exc‐6*. The genomic sequence of *fhod‐1*, including 5 kb upstream and 2 kb downstream, had been similarly cloned into pRS315‐*fhod‐1* using the GeneCATCHR method, as described previously [Mi‐Mi et al., 2012].

To produce an *exc‐6::gfp* fusion, the coding sequence for GFP, together with four synthetic introns, was amplified by PCR from pPD95.75 (provided by A. Fire, Carnegie Institute of Washington, Washington DC), and a linker encoding Gly‐Ala‐Gly‐Ala‐Gly was added to its 5' end before introduction immediately upstream of the *exc‐6* stop codon in pRS315‐*exc‐6*, again using the GeneCATCHR method. This *exc‐6::gfp* sequence was introduced into the worm transformation vector pJKL702 (a gift from J.K. Liu, Cornell University, Ithaca, NY) by standard subcloning to generate pJKL702‐*exc‐6::gfp*.

For RNAi studies, double stranded RNA‐expression vectors L4440, L4440‐*exc‐*6 (previously called L4440‐*inft‐1*), and L4440‐*fhod‐1A* were generated as previously described [Mi‐Mi et al., 2012], while L4440‐*par‐2* was a gift provided by K. Kemphues (Cornell University, Ithaca, NY).

### Worm Strains and Growth Conditions

Worms were grown under standard conditions [Brenner, 1974] at 20°C. For experiments requiring age‐synchronized populations, adult worms were allowed to lay eggs on fresh plates for 4 h before removal of the adults, yielding age‐synchronized progeny.

The strains N2 (wild type) [Brenner, 1974], ST65 (*ncIs13[ajm‐1::gfp]*) [Liu et al., 2005], and PS3517 (*unc‐119(ed4)III*) were obtained from the Caenorhabditis Genetics Center (University of Minnesota, Minneapolis, MN). The following outcrossed formin mutant strains were generated as previously described [Mi‐Mi et al., 2012]: XA8001 (*fhod‐1(tm2363)I*), XA8004 (*exc‐6(gk386)III*), XA8038 (*fhod‐1(tm2363)I;exc‐6(gk386)III*), DWP6 (*fhod‐1(tm3138)I*), and DWP10 (*fhod‐1(tm2363)I;qaIs8001[unc‐119(+) fhod‐1::gfp*). Worm strains XA8043, XA8044, and XA8045, bearing alternative *fhod‐1::gfp‐*containing genomic insertions *qaIs8002*, *qaIs8003*, and *qaIs8004*, respectively, in otherwise wild‐type backgrounds, were generated independently by microparticle bombardment [Praitis et al., 2001] of *unc‐119(ed4)III* animals with pJKL709‐*fhod‐1::gfp*, as was done for the original *fhod‐1::gfp* strain DWP3 described previously (Mi‐Mi et al., 2012), followed by six outcrosses with wild‐type N2. RNAi‐sensitive strain PD8412 (*rrf‐3(pk2042)II*) [Simmer et al., 2002] was a gift from S.S. Lee (Cornell University, Ithaca, NY).

EXC‐6::GFP‐expressing strain DWP23 (*unc‐119(ed4)III;upsIs10[unc‐119(+) exc‐6::gfp]*) was made by microparticle bombardment [Praitis et al., 2001] of pJKL702‐*exc‐6::gfp* into PS3517 worms. DWP85 (*exc‐6(gk386) unc‐119(ed4?)/(+)III;upsIs10[unc‐119(+) exc‐6::gfp]*) was generated by crossing *exc‐6::gfp* into the XA8004 background, while DWP90 (*fhod‐1(tm2363)I;unc‐119(ed4?)/(+)III;upsIs10[unc‐119(+) exc‐6::gfp]*) was generated by crossing *exc‐6::gfp* into the XA8001 background. AJM‐1::GFP‐expressing strains DWP109 (*exc‐6(gk386)III;ncIs13[ajm‐1::gfp]*), DWP141 (*fhod‐1(tm2363)I;ncIS13[ajm‐1::gfp])* and DWP139 (*fhod‐1(tm2363)I;exc‐6(gk386)III;ncIs13[ajm‐1::gfp])* were generated by appropriately crossing XA8004 and/or XA8001 with ST65.

To test whether exogenous *fhod‐1* rescues the brood size defect of XA8038, XA8038 worms were injected with a mixture of 25 ng/µL pRS315‐*fhod‐1*, mCherry‐expressing co‐injection markers (2.5 ng/µL pCJF90, 10 ng/µL pGH8, and 5 ng/µL pCJF104) [Frøkjaer‐Jensen et al., 2008], and 75 ng/µL pRS315 [Sikorski and Hieter, 1989], to produce strains DWP76 (*fhod‐1(tm2363)I;exc‐6(gk386)III;upsEx42[fhod‐1(+) Pmyo‐2::mCherry Pmyo‐3::mCherry Prab‐3::mCherry]*) and DWP78 (*fhod‐1(tm2363)I;exc‐6(gk386)III;upsEx44[fhod‐1(+) Pmyo‐2::mCherry Pmyo‐3::mCherry Prab‐3::mCherry]*). Similarly, to test whether exogenous *exc‐6* rescues XA8038 brood size defects, 25 ng/µL pRS315‐*exc‐6* was microinjected with the same co‐injection markers to generate DWP82 (*fhod‐1(tm2363)I;exc‐6(gk386)III;upsEx48[exc‐6(+) Pmyo‐2::mCherry Pmyo‐3::mCherry Prab‐3::mCherry]*) and DWP83 (*fhod‐1(tm2363)I;exc‐6(gk386)III; upsEx49[exc‐6(+) Pmyo‐2::mCherry Pmyo‐3::mCherry Prab‐3::mCherry]*).

To induce RNAi, PD8412 worms were treated for RNAi by a standard feeding method [Wang and Barr, 2005]. Briefly, cultures of *Escherichia coli* strain HT115 bearing the relevant dsRNA‐expressing plasmids were grown overnight in 2xYT with 12.5 μg/mL tetracycline and 100 μg/mL ampicillin. Overnight cultures were diluted 1:100 in fresh medium and grown three additional hours at 37°C before the addition of 0.4 mM IPTG for 3 h at 37°C to induce dsRNA production. Induced HT115 cultures were concentrated fivefold, and 150 µL of concentrated cultures were seeded onto plates. For experiments requiring RNAi against two targets, 1:1 mixtures of induced cultures for both targets were plated.

### Brood Size Assay

L4 worms were individually placed on small NGM‐OP50 plates (or plates with dsRNA‐expressing HT115 for RNAi experiments) for 12 h. Over a 4‐day period, worms were moved to a fresh plate every 12 h, and eggs and hatched progeny on the previous plate were counted. On the fifth day, worms were not moved, but the progeny on the final plate were counted on the seventh day. Ten to fifteen worms per strain or RNAi treatment were analyzed for each assay, with three repetitions of each assay.

### Microscopy and Image Analysis

To stain large numbers of animals for F‐actin and DNA, young adult worms were prepared in bulk for fluorescence microscopy as described previously [Mi‐Mi et al., 2012]. To stain the gonads of smaller numbers of animals for F‐actin and DNA, young adult worms were suspended in egg buffer (118 mM NaCl, 48 mM KCl, 2 mM CaCl_2_•2H_2_0, 2 mM MgCl_2_•6H_2_0, 25 mM HEPES, pH 7.3) supplemented with 0.2 mM levamisole (Acros Organic, Geel, Belgium), and quickly transferred to a poly‐lysine‐coated slide. Heads or tails were removed using syringe needles, allowing extrusion of the gonad. Dissected worms were fixed 15 min in egg buffer containing 2% formaldehyde and 1:250 Alexa_568_‐phalloidin (Life Technologies, Carlsbad, CA), and washed twice for 5 min with Tris‐Tween (100 mM Tris‐HCl, pH 7.4; 1 mM EDTA; 1% Tween‐20), and twice briefly in PBST (1x PBS; 0.1% Tween‐20). Washed worms were then incubated 45 min in PBST supplemented with 1:250 Alexa_568_‐phalloidin, 1 µg/mL 4′,6‐diamidino‐2‐phenylindole, dihydrochloride (DAPI), and 0.1% goat serum (Sigma‐Aldritch, St. Louis, MO), before two final brief washes in PBST and the addition of VECTASHIELD mounting medium (Vector Laboratories, Burlingame, CA).

To stain extruded gonads for AJM‐1, young adult worms were treated as above, except that worms were dissected in 1x PBS containing 0.2 mM levamisole rather than egg buffer, and following Tris‐Tween washes, the worms were washed thrice for 2 min in PBST before immersion of slides into −20°C MeOH for 5 min. Slides were then washed thrice for 2 min by immersion in PBST, and blocked for 1 h in PBST containing 1% goat serum. Blocked samples were divided into two sets. One set was incubated overnight in PBST containing 1:200 MH27 (anti‐AJM‐1) [Francis and Waterston, 1991] and 0.1% goat serum. The other set was incubated overnight in PBST containing 1:2000 goat anti‐mouse Texas Red (Rockland Immunochemicals, Pottstown, PA) and 0.1% goat serum to pre‐clear the secondary antibodies. Pre‐cleared secondary antibodies were removed from slides and kept until needed. Samples stained with MH27 were washed thrice for 2 min in PBST, and then incubated for 2 h with pre‐cleared secondary antibodies supplemented with 1 µg/ml DAPI. Slides were again washed three times for 2 min in PBST before samples were covered with VECTASHIELD.

To observe ovulations in live animals, young adult worms were paralyzed in M9 containing 0.1% tricaine (Acros Organics, Geel, Belgium) and 0.01% levamisole before mounting onto 2% agarose pads in preparation for differential interference contrast (DIC) microscopy. Images were acquired every 2 s for 30 min after the initiation of ovulation, which was identified manually by the visible breakdown of the oocyte nuclear envelope. To measure spermatheca stretch, the maximum height of the spermatheca was measured during oocyte entry.

Wide‐field fluorescence and DIC images were obtained on an Eclipse 90i research upright microscope (Nikon, Tokyo, Japan) at room temperature using a CFI Plan Apochromat 40×/NA 1.0 oil immersion objective, or a CFI Plan Apochromat violet‐corrected 60×/NA 1.4 oil immersion objective with a Cool‐SNAP HA2 digital monochrome charge‐coupled device camera (Photometrics, Tucson, AZ) driven by NIS‐Elements AR acquisition and analysis software (version 3.1; Nikon). For experiments requiring measurements of F‐actin content based on intensity of Alexa_568_‐phalloidin staining, images were consistently acquired using 45 ms exposures. Confocal images were obtained at 37°C on an SP5 laser‐scanning confocal microscope (Leica, Wetzlar, Germany) driven by LAS AF Software (version 2.2.0, build 4758; Leica), and using an HCX Plan Apochromat 63x/NA 1.4 oil lambda objective.

Images were edited linearly and colored in Photoshop CS4 (Adobe, San Jose, CA). Actin bundle widths were measured in NIS‐Elements using the measurement tool and data were transferred to Excel:mac (Microsoft Corporation, Redmond, WA) for analysis. Fluorescent phalloidin intensities were quantified on raw images (not adjusted for contrast or brightness) using ImageJ (National Institutes of Health; Bethesda, MD; http://rsb.info.nih.gov/ij/). For this, a line perpendicular to the F‐actin bundles of a single cell was drawn, and the fluorescence intensity profile was transferred to Excel:mac for averaging and further statistical analysis (below). To visualize and analyze F‐actin at cell‐cell junctions of spermathecae, XZ and YZ cross‐sections were generated from Z‐stacks of confocal images of spermathecae using the sectioning tool of IMARIS (Bitplane, Belfast, UK). Cell‐cell junctions were identified based on the presence of AJM‐1::GFP. Junctions were scored in double‐blinded samples for the presence or absence of thickened basal F‐actin bundles flanking the junctions, and for the presence or absence of F‐actin along the junction surface.

### Statistics

Numerical data in the text are expressed as mean ± one standard deviation. Graphs were made in Excel:mac or KaleidaGraph (Synergy Software, Reading, PA). For results from two groups, data were analyzed using an unpaired, two‐tail student's *t*‐test, with *P* < 0.05 considered statistically significant. For results from three or more groups, data were analyzed using one‐factor analysis of variance with Fisher's least significant difference post hoc testing. Groups were considered significantly different when the difference in their means exceeded the 95% confidence interval.

## Supporting information

Supporting Figure 1

Supporting Figure 2

Supporting Figure 3

Supporting Figure 4
