# Supplementary figures and images for: INF2‐ and FHOD‐related formins promote ovulation in the somatic gonad of C. elegans
Source: Cytoskeleton (Hoboken). 2016 Nov 9;73(12):712–28. doi: 10.1002/cm.21341 (PMC5148669; doi:10.1002/cm.21341)

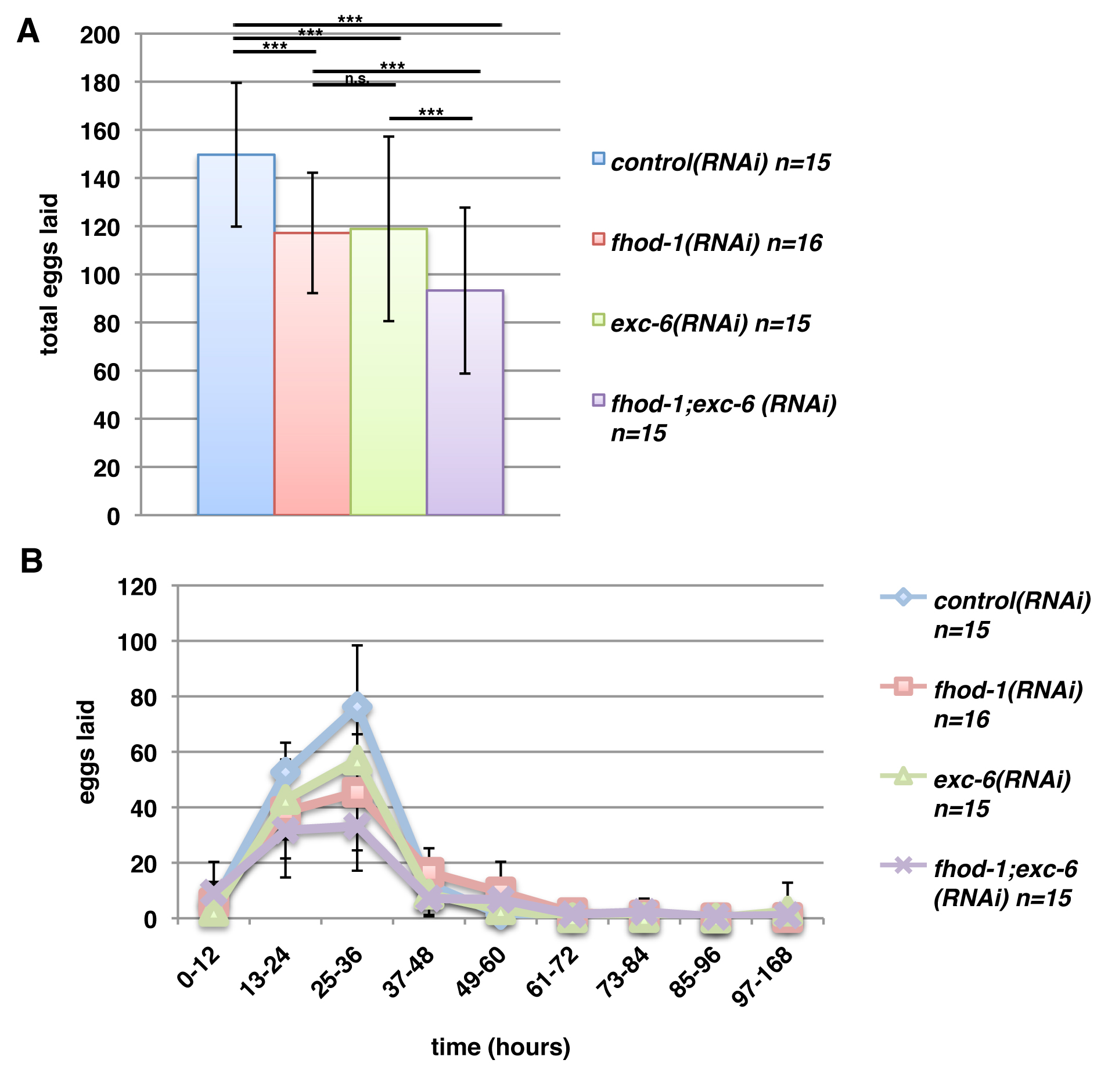

Supplement: Supplementary file 1 — Supporting Figure 1 [file CM-73-712-s001.jpg]

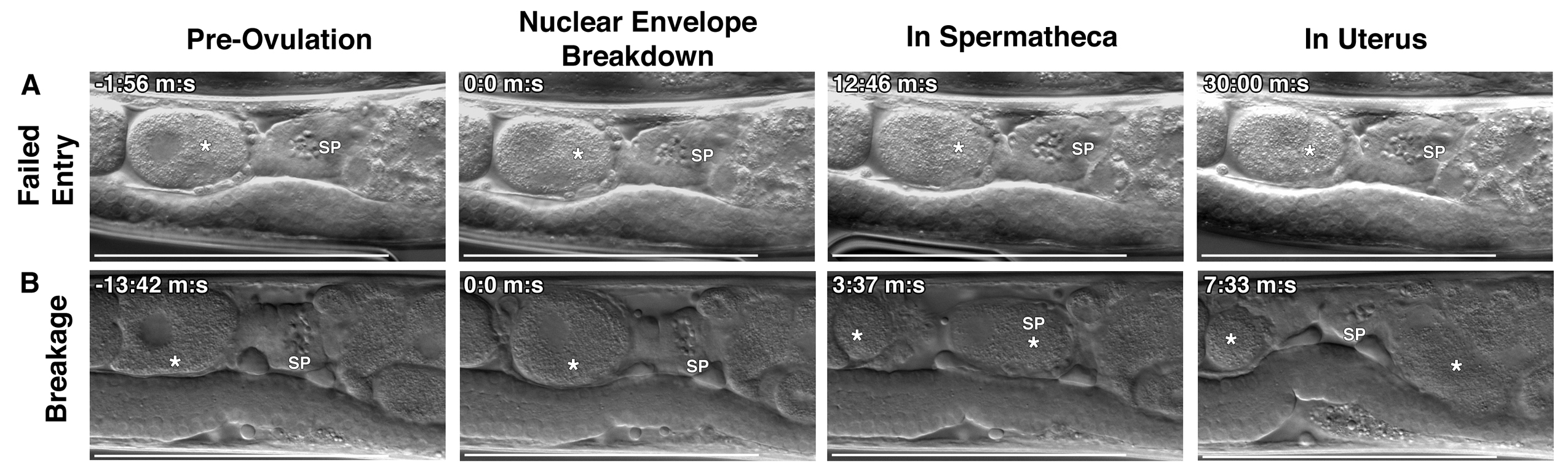

Supplement: Supplementary file 2 — Supporting Figure 2 [file CM-73-712-s002.jpg]

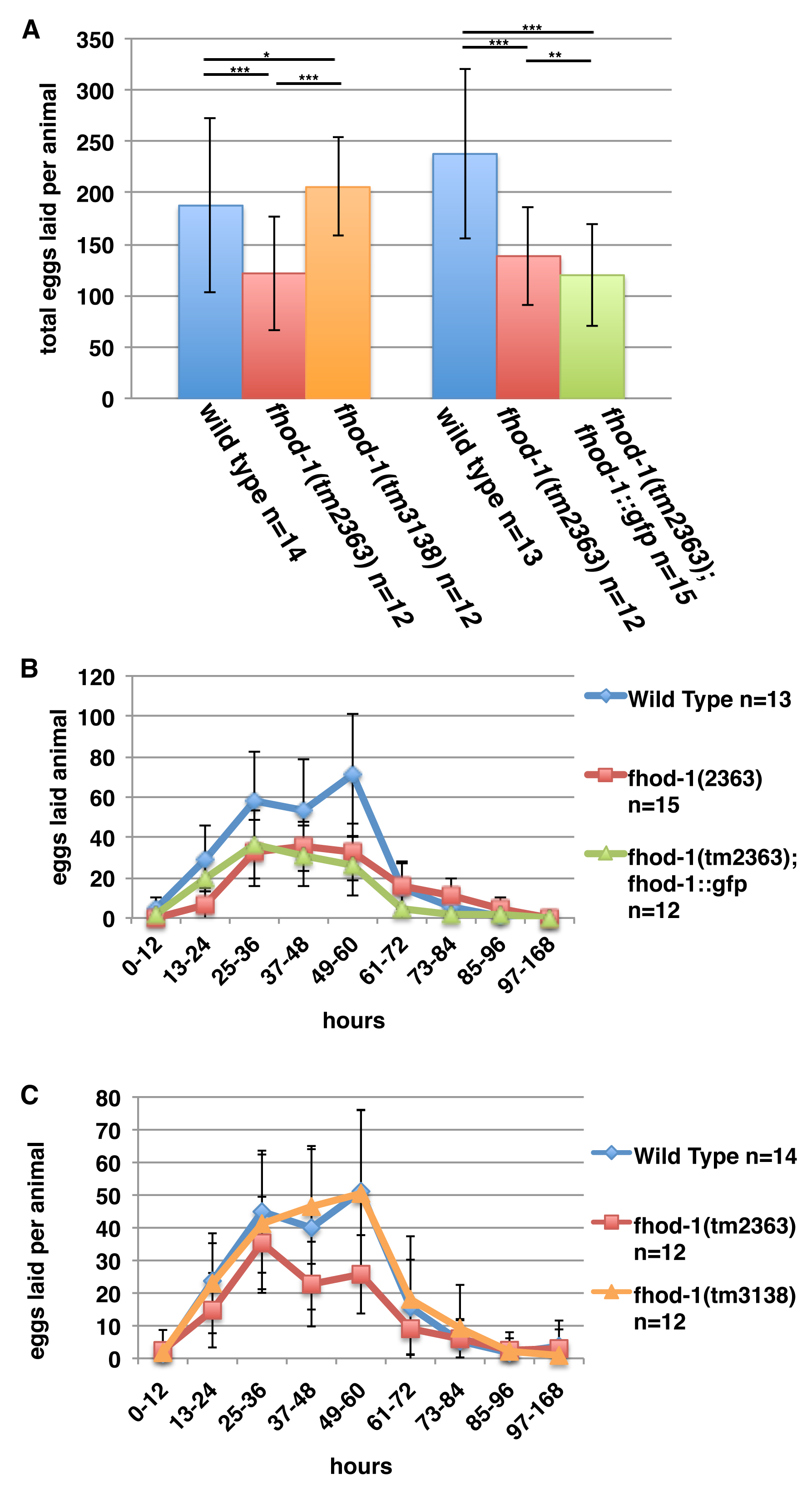

Supplement: Supplementary file 3 — Supporting Figure 3 [file CM-73-712-s003.jpg]

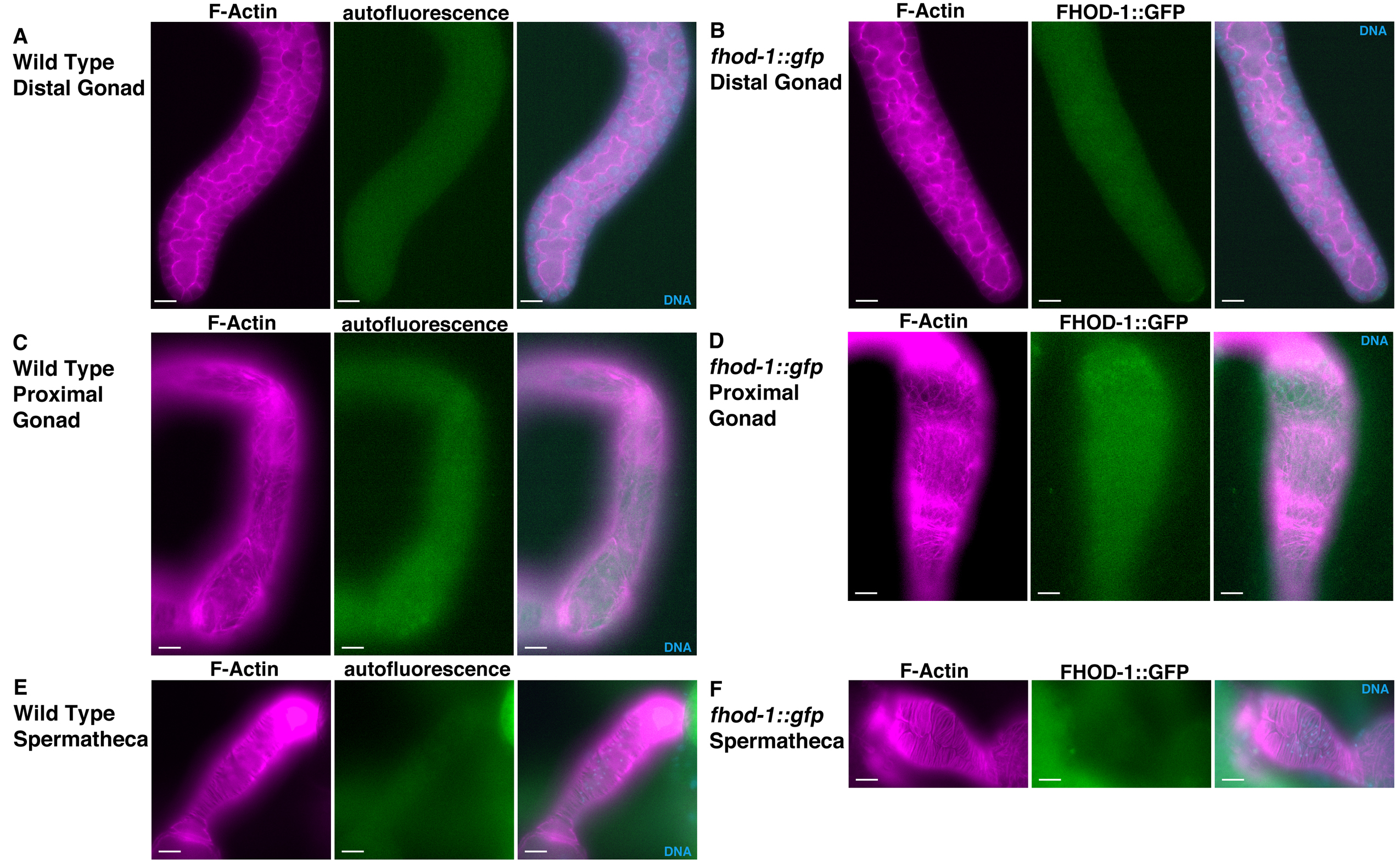

Supplement: Supplementary file 4 — Supporting Figure 4 [file CM-73-712-s004.jpg]
